# Metabolic Syndrome, BMI, and Polymorphism of Estrogen Receptor-α in Peri- and Post-Menopausal Polish Women

**DOI:** 10.3390/metabo12080673

**Published:** 2022-07-22

**Authors:** Jan Krakowiak, Dorota Raczkiewicz, Ewa Humeniuk, Artur Wdowiak, Andrzej Wróbel, Iwona Bojar

**Affiliations:** 1 Department of Social Medicine, Medical University of Lodz, Żeligowskiego 7/9 Street, 90-752 Łódź, Poland; jankrakowiak@wp.pl; 2 Department of Medical Statistics, School of Public Health, Center of Postgraduate Medical Education, Kleczewska 61/63 Street, 01-826 Warsaw, Poland; 3 Department of Psychology, Medical University of Lublin, Chodzki 7 Street, 20-400 Lublin, Poland; ewahumeniuk@umlub.pl; 4 Department of Obstetrics and Gynecology, Medical University of Lublin, 4-6 Staszica Street, 20-081 Lublin, Poland; wdowiakartur@gmail.com; 5 Second Department of Gynecology, Medical University of Lublin, Jaczewskiego 8 Street, 20-090 Lublin, Poland; wrobelandrzej@yahoo.com; 6 Department of Women’s Health, Institute of Rural Health in Lublin, ul. Jaczewskiego 2, 20-090 Lublin, Poland; iwonabojar75@gmail.com

**Keywords:** menopause, metabolic syndrome, estrogen receptor alpha polymorphism, BMI, women

## Abstract

The study aimed to investigate the association between the estrogen receptor alpha (ERα) polymorphism and the prevalence of metabolic syndrome (MetS) and obesity, as well as the coexistence of MetS and obesity, in peri- and post-menopausal Polish women. The study group consisted of 202 peri-menopausal and 202 post-menopausal women. ERα polymorphism: Xba I and Pvu II, MetS, BMI, and serum estrogen concentration were analyzed. MetS was found in 29% of the peri-menopausal women and in 21% of the post-menopausal women. BMI did not significantly differ between the peri- and post-menopausal women (≈42% were normal weight, ≈40% were overweight, and ≈18% were obese), (*p* = 0.82). Serum estrogen concentration in the peri-menopausal women was 91 ± 75 pg/mL, while that in the post-menopausal women was 17 ± 9. pg/mL, on average. Peri-menopausal women with AA and TT genotypes of the ERα polymorphism have a lower risk of obesity and MetS and the co-existence of obesity and MetS, whereas those women with the G or C allele have a higher risk of those health problems.

## 1. Introduction

Menopause is a natural process related to the loss of generative function of the ovaries, which, in turn, leads to the onset or acceleration of the development of health problems resulting from estrogen deficiency. Estrogens exhibit multidirectional protective action [1,2]. The essence of the problem is, however, more complicated than a simple hormone deficiency. A new research trend is to test the hypothesis that alpha estrogen receptor (ERα) polymorphisms may determine various effects of estrogens on the prevalence of health problems [3,4]. ERα polymorphisms affect the function of the estrogen receptor and, thus, the response of tissues to estrogen stimulation [5,6]. The dominant expression tissues of ERα are: uterus, pituitary gland, liver, hypothalamus, bones, mammary gland, cervix and vagina, adipose tissue, and skeletal muscles [7].

The genes encoding ERα have many polymorphic variants (there are about 9000 of them), among which the most important—from the clinical point of view—are two polymorphisms of the single nucleotide polymorphism (SNP) type—Xba I and Pvu II [8]. The Xba I polymorphism (A→G rs9340799) is located in intron 1 of ERα 351 bp at the 5′ end upstream of exon 2, hence its name IVS1-351 [7]. It is induced by the A→G transition [9]. Xba I is located approximately 50 bp from the Pvu II polymorphism site (T→C, rs2234693) known as IVS1-397T→C [10]. It is caused by the T→C transition in intron 1, 397 bp before the 5′ end of exon 2 [11].

Some studies have shown that ERα Xba I and Pvu II polymorphisms are associated with the risk of such diseases in menopausal women as osteoporosis, cardiovascular disease, endometriosis, neoplasms, systemic lupus erythematosus, Alzheimer’s disease, dyslipidemia, hypertension, and coronary atherosclerosis [12,13,14,15]. It is, therefore, difficult to state unequivocally which alleles are responsible for specific illnesses. It is known, however, to be a relationship dependent on ethnicity (race) [11]. AA Xba I and CC Pvu II genotypes are associated with an increased risk of osteoporosis in the Asian population, while an increased risk of osteoporosis in the Caucasian population is associated with the AA Xba I and TT Pvu II genotypes [13].

ERα polymorphisms are commonly associated with MetS and obesity in women [16,17,18,19]. Obesity (BMI > 30 kg/m^2^) is an increasingly common, multi-factor health problem. Its prevalence is increasing at an alarming rate across the globe, posing a serious public health problem. Weight gain characterized by an unfavorable redistribution of adipose tissue with an increase in visceral fat and a decrease in what is known as lean body mass can be observed in post-menopausal women. European studies of post-menopausal women showed that 28% of them had their BMI within the normal range, while 57% of them were overweight and 15% were obese [20]. In comparison to this result, 32% of American women aged 45–54-years-old were obese [21]. In a Polish study, 17% of women aged 44–66 were obese, 39% were overweight, and 44% had normal body mass; moreover, 29% had abdominal obesity, 19% had increased body fat accumulation, and 21% had high adipose tissue accumulation [22]. The most previous studies showed an association between ERα polymorphisms and obesity and obesity-related symptoms such as waist circumference and BMI in white women [23,24], in African-American women [17], and in Japanese women [25]. However, no association between ERα polymorphisms and obesity or obesity parameters was demonstrated in Swedish women [26] and in the Chinese population [27].

The results of some studies showed that polymorphisms are also associated with MetS and the components of MetS [17,24,28,29]. Recognizing MetS requires a diagnosis of at least three of the following criteria: abdominal obesity, high triglycerides (TG), low HDL-cholesterol, high blood pressure, and high fasting blood glucose (FBG) [30]. MetS is spread widely throughout the population and its prevalence is continually rising, causing serious health problems. According to a cross-sectional population study in Brazil, the prevalence of MetS in menopausal women was 56.9% [31]. In a study on postmenopausal women in Poland, the prevalences of MetS were 70% and 22% in rural and urban areas, respectively [32]. ERα polymorphisms were also associated with changes in BMI, waist circumference, and components of MetS [18,33,34,35]. An analysis of the literature showed that ERα polymorphisms are important for the influence of estrogens on the functioning of the body and may implicate the development of many pathological health problems, including obesity and MetS in perimenopausal women. The study aimed to investigate the association between the ERα polymorphism and the prevalence of MetS and obesity, as well as the coexistence of MetS and obesity, in peri- and post-menopausal Polish women.

## 2. Results

### 2.1. Distribution of ERα Polymorphisms Xba I and Pvu II in the Peri- and Post-Menopausal Women

Table 1 presents the distribution of the ERα polymorphisms: Xba I and Pvu II in the peri- and post-menopausal women. These two groups of women did not significantly differ in respect of these two polymorphisms.

### 2.2. Prevalence of Metabolic Syndrome by ERα Polymorphisms Xba I and Pvu II in the Peri- and Post-Menopausal Women

Table 2 presents the prevalence of MetS in peri- and post-menopausal women in total and with various genotypes of the ERα polymorphisms: Xba I and Pvu II. MetS was found in 29% of the peri-menopausal women and in 21% of the post-menopausal women. The prevalence of MetS in peri-menopausal women was associated with both Xba I and Pvu II. It was the least common in the peri-menopausal women with AA and TT genotypes, but it was not associated with Xba I and Pvu II in the post-menopausal women.

### 2.3. BMI by ERα Polymorphisms Xba I and Pvu II in the Peri- and Post-Menopausal Women

Table 3 presents the BMI of the peri- and post-menopausal women in total and with various genotypes of the ERα polymorphisms: Xba I and Pvu II. BMI did not significantly differ between the peri- and post-menopausal women (≈42% were normal weight, ≈40% were overweight, and ≈18% were obese). In the peri-menopausal women, BMI was not associated with Xba I, while it was almost significant with Pvu II. The percentage of obese women was significantly higher in CC than in TT and TC genotypes. In the post-menopausal women, BMI was not associated with Xba I and Pvu II.

### 2.4. Serum Estrogen Concentration by ERα Polymorphisms Xba I and Pvu II in the Peri- and Post-Menopausal Women

Serum estrogen concentration in the peri-menopausal women was 91.1 ± 74.8 pg/mL, while that in the post-menopausal women was 17.3 ± 9.1 pg/mL, on average. Both in the peri- and post-menopausal women, serum estrogen concentration was not associated with Xba I and Pvu II.

Table 4 presents the serum estrogen concentration in peri- and post-menopausal women by the prevalence of MetS and by BMI. The peri-menopausal women with MetS had a significantly lower serum estrogen concentration than those without MetS. In the post-menopausal women, serum estrogen concentration was not associated with the presence of MetS. Both in the peri- and post-menopausal women, serum estrogen concentration was not associated with BMI.

### 2.5. Do Odds of Metabolic Syndrome, Overweight, or Obesity Depend on ERα Polymorphisms Xba I and Pvu II and Serum Estrogen Concentration in the Peri- and Post-Menopausal Women?

Table 5 presents the results of logistic regression analysis for MetS (versus no MetS), as well as for overweight or obesity versus normal weight, separately in the peri-menopausal women and in the post-menopausal women. In the model for MetS (versus no MetS) in the peri-menopausal women, the odds of MetS were approximately 6.5-fold higher in the women with GG compared to AA and 3.5-fold higher in the women with TC compared to TT, and they decreased by 0.6% with increasing serum estrogen concentration by 1 pg/mL, on average. In such a model for MetS in the post-menopausal women, no significant association was found. In the model for overweight or obesity (versus normal weight) in the peri-menopausal women, the odds of overweight or obesity were ≈3-fold higher in the women with TC compared to TT; however, associations with XBA I and with serum estrogen concentration were not found. In such a model for overweight or obesity in the post-menopausal women, no significant association was found.

### 2.6. Coexistence of Metabolic Syndrome and BMI by ERα Polymorphisms Xba I and Pvu II in the Peri- and Post-Menopausal Women

Table 6 presents the prevalence of coexisting MetS and BMI in peri- and post-menopausal women in total and in those women with various genotypes of the ERα polymorphisms: Xba I and Pvu II. The coexistence of MetS and BMI did not significantly differ between the peri- and post-menopausal women. Overweight and MetS were found in 16% of the peri-menopausal women and in 9.5% of the post-menopausal women; obesity and MetS were found in 10% and 8%, respectively. In the peri-menopausal women, the coexistence of MetS and BMI was associated with Xba I and Pvu II. The percentage of overweight or obese women with MetS was significantly higher in AG and GG than in AA genotypes. The percentage of overweight or obese women with MetS was significantly higher in TC and CC than in TT genotypes. In the post-menopausal women, the coexistence of MetS and BMI was not associated with Xba I and Pvu II.

## 3. Discussion

We investigated the association between Xba I and Pvu II ERα polymorphisms and the prevalence of MetS, obesity, and the coexistence of MetS and obesity in the peri- and post-menopausal women in Poland. We observed that ERα polymorphisms may be associated with the coexistence of MetS and obesity. To our knowledge, this is the first study on this topic.

Previous scientific studies showed different distributions of Xba I and Pvu II ERα polymorphisms in different populations. The results of our study are consistent with Mysliwska’s study in which 20% of the white female population are homozygous CC, slightly over 20% are homozygous TT, and most are heterozygous TC [36]. Similarly, Koch and Shearman found that the TC genotype was the most common, and the CC Pvu II genotype was the least common [37,38]. In the study of post-menopausal white women, Lamon-Fava observed that among the women, the TC genotype of the Pvu II polymorphism was the most common, whereas CC was the least common. The same study showed that with regard to the Xba I ERα polymorphism, most women had the AG genotype, and the least women had GG [39]. Lian reported that the AG Xba I and TC Pvu II genotypes were the most common among Europeans, while Dai found that the AA Xba I and TC Pvu II genotypes were the most common in Asians. Interestingly, both researchers reported that among both the white and Asian populations, the least common genotypes were GG Xba I and CC Pvu II [40,41,42,43].

Results similar to ours, regarding overweight and obesity in the population of menopausal women, were obtained in other studies [44,45]. In the female population in Turkey, it was observed that a significantly reduced risk of obesity in menopausal women was found in carriers of the AG genotype and in carriers of the G allele [18]. Another study indicated that the G allele was associated with a lower BMI and a lower waist circumference in African-American families [17], and that the prevalence of obesity was higher in the women with TC and TT than in the women with CC [46]. Carrying the G allele in the homozygous or heterogenous form (genotypes GG or AG) is associated with a higher BMI and a higher waist circumference [47]. In the Gomes-Rochette study, the TC genotype was associated with a lower level of body fat and a higher level of lean mass and water in the body, whereas the AG genotype was associated with a higher BMI [33].

Earlier studies showed that the less common G allele of the Xba I polymorphism was more commonly observed in the patients with MetS than in the control group (AG and GG were found in 55% and 30% of the patients, respectively, and in 55.3% and 30% of the control group, respectively) [47].

A study by Yang et al. showed that neither the Pvu II nor Xba I polymorphisms are associated with a risk of MetS [29]. In other studies, the T Pvu II allele was associated with the risk of hyperlipidemia in post-menopausal Chinese women [48], with increased amounts of small LDL particles [49], with decreased HDL-cholesterol and increased TG serum concentrations, as well as with increased susceptibility to lipid metabolism disorders [39]. A recent study conducted on Brazilian post-menopausal women showed no effect of the Pvu II polymorphism on total cholesterol, LDL-cholesterol, HDL-cholesterol, and TG in the patients with dyslipidemia, while the Xba I polymorphism was associated with changes in TG and total lipids, mainly in obese and overweight women [33]. On the contrary, an Egyptian study found that both Pvu II and Xba I are associated with increased levels of TG, total cholesterol, and LDL-cholesterol [50]. Carriers of the homozygous or heterozygous G allele (GG or AG genotypes) had higher systolic and diastolic blood pressure, FBG, fasting serum insulin, as well as total cholesterol and LDL-cholesterol [33,47].

A study by Toaima et al. showed that the patients with the CC genotype had better glycemic control than the patients with other genotypes [46]. A meta-analysis conducted in 2018 and consisting of eight studies showed that it was polymorphism Pvu II, but not polymorphism Xba I, that was associated with type 2 diabetes mellitus (T2DM) [43]. The C allele of the Pvu II polymorphism showed a protective role in T2DM in the Chinese population [7], while the G allele of the Xba I polymorphism was associated with a reduced risk of T2DM in the Caucasian population [29].

A recent study found that the presence of a less common G allele favored lower waist circumference and BMI compared to a more common A allele, regardless of age, smoking habits, alcohol consumption, physical activity, diabetes, or menopausal status [18]. Women with AG Xba I had significantly higher TG and total cholesterol serum concentrations than women with other genotypes of this polymorphism, referring to both obese and older women [33].

Although the pathogenesis of obesity and MetS are thought to be correlated with many factors, genetics is considered as one of the significant determinants. Studies using the estrogen receptor ERα knockout mice have demonstrated that ERα plays an essential role in estrogen-mediated metabolic regulation [51]. Therefore, changing estrogen levels during the menopausal transition in women may influence peri and postmenopausal metabolic changes. It has been confirmed that the Pvu II polymorphism may affect the expression levels of mRNA, thus altering the protein expression [52]. In addition, a possible functional mechanism that is attributed to PvuII and Xba I is that these polymorphisms could change the expression of the ERα gene by alternating the binding of transcription factors and influence the alternative splicing of the ERα gene [53]. Furthermore, the ERα gene polymorphism may affect the levels of plasma adiponectin in postmenopausal women [54], which plays a wide-ranging role in metabolic processes, such as food intake and metabolism of carbohydrates and lipids [55].

The limitation of our study is the number of respondents and the number of polymorphisms because of the high costs of genetic studies. Because this study was based on cross-sectional data, the cause-and-effect relationship should not be assumed. It would be worth conducting further research such as cause–effect and longitudinal studies on the association between the ERα polymorphism and MetS and BMI changes in the same women in different periods of their lives: pre-, peri-, and post-menopause. In the future, a larger sample size, more accurate sample information, and a more rigorous and sensible study design are needed to comprehensively validate an association between the ERα polymorphism and MetS and BMI. It is necessary to define the molecular mechanisms thanks to the ERα gene polymorphism affecting MetS and obesity in the peri- and post-menopausal women group. This requires further research on a larger sample.

## 4. Materials and Methods

### 4.1. Study Group

The data were collected in the years 2017–2020 in the Institute of Rural Health in Lublin, Poland. An advertising and promotion campaign about this study was conducted both via the internet and leaflets that were distributed to various places and workplaces that employed women. Women volunteered for research in the Institute and those who met the inclusion criteria were selected for the study. Informed consent for participation in the study was obtained from all the women. The study was approved by the Ethics Committee of the Institute of Rural Medicine in Lublin, Poland.

Based on the STRAW criteria [56,57], 2 groups of women according to their reproductive status were included in the study: 202 peri-menopausal women and 202 post-menopausal women. The examined peri-menopausal women were aged 44–60, mean age 49.5 ± 3.2 years; those during the post-menopausal period were aged 46–66, mean age 56.2 ± 3.3 years. Women who used hormone-replacement therapy were not included in the study. Blood pressure was measured in the morning sitting down after 15 min of rest with a standardized blood pressure monitor.

The examined women were weighted, their height and waist circumference were measured, and their BMI was calculated. Based on this, they were qualified as normal weight, overweight, or obese.

### 4.2. Laboratory Blood Tests

Blood samples were taken from the examined women to carry out the following laboratory tests: Total cholesterol, HDL-cholesterol, TG, estrogen, and follicle stimulating hormone. Blood samples were immediately taken to a certified laboratory “ALAB”. LDL-cholesterol = Total cholesterol—HDL-cholesterol—1/5 triglycerides was calculated.

### 4.3. Determining the Metabolic Syndrome

The presence of MetS was determined in the examined women according to the International Diabetes Federation [58]. MetS was defined as the coexistence of at least 3 of the following 5 risk factors: Waist circumference ≥ 80 cm; TG ≥ 150 mg/dL or treatment for dyslipidemia; HDL-cholesterol < 50 mg/dL or treatment for dyslipidemia; Systolic blood pressure ≥ 130 mm Hg and/or diastolic blood pressure ≥ 85 mm Hg or antihypertensive therapy; Fasting glucose ≥ 100 mg/dL or hypoglycemic treatment.

### 4.4. DNA Isolation

Genomic DNA isolation was derived from 0.2 mL of human blood by QIAamp DNA Blood Mini Kit (Qiagen, Düsseldorf, Germany), as per the producer’s instructions. The amount and purity of the extracted DNA were measured using the NanoDrop spectrophotometer.

### 4.5. ERα Polymorphisms

Polymorphisms of ERα were determined using the restriction fragment length polymorphism (RFLP-PCR) method. PCR reaction was performed in a total amount of 50 μL containing: 1 U (1 μL) of DNA polymerase (BioTools Inc., Jupiter, FL, USA), 1·PCR buffer (5 μL) containing 15 mM MgCl2 (Biotools), 2.5 μL of 2 mM dNTPs (final concentration 0.1 mM) (Fermentas, Vilnius, Lithuania), 1 μL of 10 μM of each of the 2 primers, 34.5 μL of nuclease-free water (Applied Biosystems Inc., Waltham, MA, USA), and 5 μL of genomic DNA. The reactions were performed in a C1000 Thermal Cycler (Bio-Rad, Hercules, CA, USA) and consisted of the initial denaturation (3 min at 95 °C), and 30 cycles, each of which included the proper denaturation (30 s at 95 °C), primers annealing (50 s at 62 °C), elongation (50 s at 72 °C), and the final elongation (7 min at 72 °C). Electrophoresis was performed in 2% agarose gel under standard conditions. The products of PCR (1372 bp) were digested overnight at 37 °C using 2 separate restriction enzymes for determining the polymorphisms: Pvu II (c.454-397 T→C) and Xba I (c.454-351 A→G). The products of restriction were electrophoresed in 2.5% agarose gel.

The alleles of the Xba I polymorphism were defined as A and G: heterozygote AG (fragments: 1372 bp, 936 bp, and 436 bp), homozygote GG (fragment: 1372 bp), and homozygote AA (fragments: 936 bp and 436 bp). The alleles of the Pvu II polymorphism were defined as T and C: heterozygote TC (fragments: 1372 bp, 982 bp, and 390 bp), homozygote TT (982 bp and 930 bp), and homozygote CC (1372 bp).

### 4.6. Statistical Methods

The statistical analyses were conducted using SPSS software. The mean ($\bar{x}$) and standard deviation (SD) were estimated for continuous variables, as well as absolute numbers (n) and percentages (%) of the occurrence of items for categorical variables. Pearson’s chi-square test was used to compare genotypes between peri- and post-menopausal women, as well as to compare the prevalence of MetS, BMI, and the coexistence of MetS and BMI between genotypes, separately in peri-menopausal women and in post-menopausal women. Student’s t-test was used to compare serum estrogen concentrations between women with and without MetS, separately in peri-menopausal women and in post-menopausal women. The F test of analysis of variance was used to compare serum estrogen concentrations between three genotypes, as well as to compare serum estrogen concentrations between women with normal weight, overweight, and obesity, separately in peri-menopausal women and in post-menopausal women. We also estimated logistic regression models for MetS (versus no MetS) and for overweight or obesity (versus normal weight). Predictors were: Xba I and Pvu II ERα polymorphisms and serum estrogen concentration. All the logistic regression analyses were conducted separately for peri-menopausal women, and separately for post-menopausal women. The significance level was assumed to be 0.05.

## 5. Conclusions

The prevalence of Xba I and Pvu II ERα polymorphisms in the studied population of peri- and post-menopausal women was varied. The carrier of alleles G and C, especially in homozygotes, may be a risk factor for obesity in peri-menopausal women. In peri-menopausal women, there is a statistically significant correlation between genotype carried and the prevalence of the metabolic syndrome. The carrying of alleles G and C is a risk factor for metabolic syndrome in peri-menopausal women. An association between the ERα polymorphism genotype and the coexistence of obesity and MetS in the group of peri-menopausal women was observed. Peri-menopausal women with AA and TT genotypes of the ERα polymorphism have a lower risk of obesity and MetS and the co-existence of obesity and MetS. Those women with G or C alleles have a higher risk of those health problems. The presented results may provide information on the importance of genetic factors in the development of MetS and obesity in peri- and post-menopausal women. A better understanding of this association can contribute to the implementation of an appropriate screening and prevention strategy to maintain the health of menopausal women. It can also become the basis for the development of innovative therapeutic interventions.

## Figures and Tables

**Table 1:** Polymorphism of estrogen receptor alpha: Xba I and Pvu II in peri- and post- menopausal women.

Polymorphism of Estrogen Receptor Alpha	Genotypes	Peri-Menopausal Women (n = 202)	Post-Menopausal Women (n = 202)	*p* ^1^
Xba I	AA	95 (47.0)	86 (42.6)	0.18
	AG	78 (38.6)	95 (47.0)	
	GG	29 (14.4)	21 (40.0)	
Pvu II	TT	62 (30.7)	54 (26.7)	0.68
	TC	95 (47.0)	101 (50.0)	
	CC	45 (22.3)	47 (23.3)	

Results are presented as *n* (%). ^1^ *p* for chi-square test. The 3 genotypes in each polymorphism in each women group make up 100%.

**Table 2:** Prevalence of metabolic syndrome in peri- and post-menopausal women in total and by polymorphism of estrogen receptor alpha: Xba I and Pvu II.

Polymorphism of Estrogen Receptor Alpha	Genotypes	Peri-Menopausal Women		Post-Menopausal Women	
Total		59 (29.2)	*p* ^1^	43 (21.3)	*p* ^1^
Xba I	AA	18 (19.0)	0.010	20 (23.3)	0.67
	AG	30 (38.5)		20 (21.1)	
	GG	11 (37.9)		3 (14.3)	
Pvu II	TT	8 (12.9)	0.001	16 (29.6)	0.17
	TC	38 (40.0)		20 (19.8)	
	CC	13 (28.9)		7 (14.9)	

Results are presented as *n* (%). ^1^ *p* for chi-square test to compare prevalence of metabolic syndrome between genotypes.

**Table 3:** BMI in peri- and post-menopausal women in total and by polymorphism of estrogen receptor alpha: Xba I and Pvu II.

Polymorphism of Estrogen Receptor Alpha	Genotypes	BMI	Peri-Menopausal Women		Post-Menopausal Women	
Total		normal weight ^1^	87 (43.1)	*p* ^4^	81 (40.1)	*p* ^4^
		overweight ^2^	80 (39.6)		83 (41.1)	
		obesity ^3^	35 (17.3)		38 (18.8)	
Xba I	AA	normal weight	39 (41.1)	0.46	28 (32. 6)	0.12
		overweight	39 (41.1)		40 (46.5)	
		obesity	17 (17. 9)		18 (20.9)	
	AG	normal weight	36 (46.2)		40 (42.1)	
		overweight	32 (41.0)		39 (41.1)	
		obesity	10 (12.8)		16 (16.8)	
	GG	normal weight	12 (41.4)		13 (61.9)	
		overweight	9 (31.0)		4 (19.1)	
		obesity	8 (27.6)		4 (19.1)	
Pvu II	TT	normal weight	30 (48.4)	0.06	19 (35.2)	0.83
		overweight	23 (37.1)		23 (42.6)	
		obesity	9 (14.5)		12 (22.2)	
	TC	normal weight	35 (36.8)		41 (40.6)	
		overweight	46 (48.4)		43 (42.6)	
		obesity	14 (14.7)		17 (16.8)	
	CC	normal weight	22 (48. 9)		21 (44.7)	
		overweight	11 (24.4)		17 (36.2)	
		obesity	12 (26.7)		9 (19.2)	

^1^ Normal weight if BMI 18.5–24.9; ^2^ overweight BMI 25.0–29.9; ^3^ obesity BMI at least 30.0 kg/m^2^. Results are presented as *n* (%). ^4^ *p* for chi-square test to compare BMI between genotypes. The 3 BMI groups in each genotype in each polymorphism in each women group make up 100%.

**Table 4:** Serum estrogen concentration (pg/mL) in peri- and post-menopausal women by prevalence of metabolic syndrome and by BMI.

Variable	Category	Peri-Menopausal Women		Post-Menopausal Women	
		Estrogen $\bar{x}\;{}^{4}$ ± $\mathrm{SD}\;{}^{5}$	*p* ^6^	Estrogen $\bar{x}$ ± SD	*p* ^6^
Metabolic syndrome	yes	66.5 ± 60.5	0.045	17.4 ± 6.6	0.94
	no	107.1 ± 79.4		17.3 ± 9. 7	
BMI	normal weight ^1^	98.2 ± 83.1	0.30	17.8 ± 10.2	0.43
	overweight ^2^	90.6 ± 71.6		16.3 ± 7.9	
	obesity ^3^	74.8 ± 57.3		18.3 ± 9.2	

^1^ Normal weight if BMI 18.5–24.9; ^2^ overweight BMI 25.0–29.9; ^3^ obesity BMI at least 30.0 kg/m^2^. ^4^ $\bar{x}$– mean. ^5^ *SD*—standard deviation. ^6^ *p* for Student’s *t*-test to compare estrogen between women with and without metabolic syndrome or *p* for analysis of variance *F* test to compare estrogen between women with normal weight, overweight, and obesity.

**Table 5:** Logistic regression models for metabolic syndrome, and overweight or obesity against polymorphism of estrogen receptor alpha: Xba I and Pvu II, and serum estrogen concentration in peri- and post-menopausal women.

Dependent Variable	Independent Variable	Category or Unit	Peri-Menopausal Women			Post-Menopausal Women		
			OR ^4^	*p* ^5^	95%CI ^6^	OR ^4^	*p* ^5^	95%CI ^6^
Metabolic syndrome (vs. no metabolic syndrome)	Xba I	AA	ref. ^5^	-	-	ref.	-	-
		AG	1.73	0.24	0.69–4.31	1.89	0.29	0.59–6.07
		GG	6.67	0.044	1.05–42.35	1.55	0.66	0.23–10.61
	Pvu II	TT	ref.	-	-	ref.	-	-
		TC	3.52	0.022	1.20–10.34	0.36	0.09	0.11–1.19
		CC	0.59	0.57	0.09–3.71	0.25	0.09	0.05–1.23
	Serum estrogen concentration	pg/mL	0.99	0.024	0.99–1.00	1.00	0.856	0.97–1.04
Overweight ^2^ or obesity ^3^ (vs. normal weight ^1^)	Xba I	AA	ref.	-	-	ref.	-	-
		AG	0.47	0.10	0.19–1.15	0.53	0.15	0.22–1.27
		GG	1.27	0.75	0.30–5.43	0.36	0.11	0.15–1.22
	Pvu II	TT	ref.	-	-	ref.	-	-
		TC	2.91	0.026	1.14–7.44	1.30	0.60	0.50–3.39
		CC	0.99	0.99	0.26–3.81	2.09	0.25	0.60–7.33
	Serum estrogen concentration	pg/mL	1.00	0.16	0.99–1.00	1.00	0.85	0.97–1.03

^1^ Normal weight if BMI 18.5–24.9; ^2^ overweight BMI 25.0–29.9; ^3^ obesity BMI at least 30.0 kg/m^2^. ^4^ OR—odds ratio; ^5^ *p*-value for logistic regression parameters; ^5^ ref.—reference category; ^6^ CI—confidence interval.

**Table 6:** Metabolic syndrome and BMI in peri- and post-menopausal women in total and by polymorphism of estrogen receptor alpha: Xba I and Pvu II.

Polymorphism of Estrogen Receptor Alpha	Genotypes	Metabolic Syndrome	BMI	Peri-Menopausal Women		Post-Menopausal Women	
Total		no	normal weight ^1^	80 (39.6)	*p* ^4^	74 (36.6)	*p* ^4^
			overweight ^2^	48 (23.8)		64 (31.7)	
			obesity ^3^	15 (7.4)		21 (10.4)	
		yes	normal weight	7 (3.5)		7 (3.5)	
			overweight	32 (15.8)		19 (9.4)	
			obesity	20 (9.9)		17(8.4)	
Xba I	AA	no	normal weight	38 (40.0)	0.025	25 (29.1)	0.43
			overweight	29 (30.5)		30 (34.9)	
			obesity	10 (10.5)		11 (12.8)	
		yes	normal weight	1 (1.1)		3 (3.5)	
			overweight	10 (10.5)		10 (11.6)	
			obesity	7 (7.4)		7 (8.1)	
	AG	no	normal weight	30 (3.5)		36 (37. 9)	
			overweight	16 (20.5)		31 (32.6)	
			obesity	2 (2.6)		8 (8.4)	
		yes	normal weight	6 (7.7)		4 (4.2)	
			overweight	16 (20.5)		8 (8.4)	
			obesity	8 (10.3)		8 (8.4)	
	GG	no	normal weight	12 (41.4)		13 (61.9)	
			overweight	3 (10.3)		3 (14.3)	
			obesity	3 (10.3)		2 (9.5)	
		yes	normal weight	0 (0.0)		0 (0.0)	
			overweight	6 (20.7)		1 (4.8)	
			obesity	5 (17.2)		2 (9.5)	
Pvu II	TT	no	normal weight	29 (46.8)	0.004	17 (31.5)	0.70
			overweight	18 (29.0)		15 (27.8)	
			obesity	7 (11.3)		6 (11.1)	
		yes	normal weight	1 (1.6)		2 (3.7)	
			overweight	5 (8.1)		8 (14.8)	
			obesity	2 (3.2)		6 (11.1)	
	TC	no	normal weight	29 (30.5)		36 (35.6)	
			overweight	25 (26.3)		35 (34.7)	
			obesity	3 (3.2)		10 (9.9)	
		yes	normal weight	6 (6.3)		5 (5.0)	
			overweight	21 (22.1)		8 (7.9)	
			obesity	11 (11.6)		7 (6.9)	
	CC	no	normal weight	22 (48.9)		21 (44.7)	
			overweight	5 (11.1)		14 (29.8)	
			obesity	5 (11.1)		5 (10.6)	
		yes	normal weight	0 (0.0)		0 (0.0)	
			overweight	6 (13.3)		3 (6.4)	
			obesity	7 (15.6)		4 (8.5)	

^1^ Normal weight if BMI 18.5–24.9; ^2^ overweight BMI 25.0–29.9; ^3^ obesity BMI at least 30.0 kg/m^2^. Results are presented as *n* (%). ^4^ *p* for chi-square test to compare coexistence of metabolic syndrome and BMI between genotypes. Coexistence of metabolic syndrome and 3 BMI groups in each genotype in each polymorphism in each women group make up 100%.

## Data Availability

The datasets generated during the current study are available from the corresponding author on reasonable request. The data are not publicly available, due to privacy restrictions.
